# The Effect of 17α-Ethynylestradiol on Steroidogenesis and Gonadal Cytokine Gene Expression Is Related to the Reproductive Stage in Marine Hermaphrodite Fish

**DOI:** 10.3390/md11124973

**Published:** 2013-12-11

**Authors:** Isabel Cabas, Elena Chaves-Pozo, Alicia García-Alcázar, José Meseguer, Victoriano Mulero, Alfonsa García-Ayala

**Affiliations:** 1 Department of Cell Biology and Histology, Faculty of Biology, Regional Campus of International Excellence “Campus Mare Nostrum”, University of Murcia, Murcia 30100, Spain; E-Mails: icabas@um.es (I.C.); meseguer@um.es (J.M.); vmulero@um.es (V.M.); 2 Centro Oceanográfico de Murcia, Instituto Español de Oceanografía (IEO), Carretera de la Azohía s/n, Puerto de Mazarrón, Murcia 30860, Spain; E-Mails: elena.chaves@mu.ieo.es (E.C.-P.); alicia.garcia@mu.ieo.es (A.G.-A.)

**Keywords:** 17α-ethynylestradiol, steroidogenic enzymes, testis, immune-related molecules, gilthead seabream

## Abstract

Pollutants have been reported to disrupt the endocrine system of marine animals, which may be exposed through contaminated seawater or through the food chain. Although 17α-ethynylestradiol (EE_2_), a drug used in hormone therapies, is widely present in the aquatic environment, current knowledge on the sensitivity of marine fish to estrogenic pollutants is limited. We report the effect of the dietary intake of 5 µg EE_2_/g food on different processes of testicular physiology, ranging from steroidogenesis to pathogen recognition, at both pre-spermatogenesis (pre-SG) and spermatogenesis (SG) reproductive stages, of gilthead seabream (*Sparus aurata* L.), a marine hermaphrodite teleost. A differential effect between pre-SG and SG specimens was detected in the sex steroid serum levels and in the expression profile of some steroidogenic-relevant molecules, vitellogenin, double sex- and mab3-related transcription factor 1 and some hormone receptors. Interestingly, EE_2_ modified the expression pattern of some immune molecules involved in testicular physiology. These differences probably reflect a developmental adjustment of the sensitivity to EE_2_ in the gilthead seabream gonad.

## 1. Introduction

A wide variety of chemicals discharged from industrial and municipal sources have been reported to disrupt the endocrine system of marine animals, which may be exposed via the food chain or directly through contaminated seawater [1]. Recent evidence suggests that endocrine disruption as a consequence of estrogen exposure may have very serious consequences for the wild fish populations [2]. An inevitable consequence of the increasing consumption of pharmaceuticals is an increased level of contamination of surface and ground waters by these biologically active drugs, accompanied by a greater potential for adverse effects on aquatic wildlife [3]. Estrogenic pollutants are adsorbed to the sediment and could be included in the benthic food chain, in the end affecting pelagic fish [4]. Moreover, it has been demonstrated that contaminated marine sediments alter the expression of genes that are biomarkers for fish endocrine disruption [5]. 17α-Ethynylestradiol (EE_2_), a pharmaceutical compound used in oral contraceptives and hormone replacement therapy with a strong affinity for estrogen receptors (ER) [6], has a widespread presence in the aquatic environment [7]. It reaches concentrations of 0.5 to 62 ng/L in European sewage and surface waters [8,9]. Several fish species have been bath-exposed to environmental concentrations of EE_2_ (up to 10 ng/L) to ascertain any effect on reproduction [10]. Importantly, long term exposure to environmental estrogens has been shown to have an impact on the severity of the subsequent effects on reproductive development and fertility: Concentrations of EE_2_ as low as 4–6 ng/L are able to affect the fertility of the F1 generation, but not the fertility of the F0 generation [10]. Moreover, several food-web models have predicted the bioaccumulation of EE_2_ throughout the food chain [11] and this ability should not be underestimated. The determination of the impact of even low concentrations of EE_2_ on fish reproduction is therefore advisable.

Reproduction in fish is subject to hormonal regulation by gonadal steroids [12,13]. Dihydrotestosterone (DHT) is one of the most physiologically important androgens in many male vertebrates [14], with the exception of teleost fish, in which testosterone (T) and 11-ketotestosterone (11KT) are generally considered the major and most potent circulating male androgens [15]. T levels increase in both females and males during gonadal development, while 11KT is considered to be the dominant androgen in males [13,15]. While 17β-estradiol (E_2_) has been considered to be the main sex steroid of female fish, recent studies have suggested that estrogens are “essential” for normal male reproduction [16,17,18]. However, little is known about the local immune regulation that takes place in the fish testis that provides protection for the developing male germ cells, while permitting qualitatively normal inflammatory responses and protection against infection [19].

The gilthead seabream (*Sparus aurata* L.) is a seasonally breeding, marine, protandrous hermaphrodite teleost. The specimens are male during the first 2 years of life and subsequently change into females. During the male phase, the bisexual gonad has functional testicular and non-functional ovarian areas [20,21]. Therefore, the gonad of this species could be considered a complex model in which both ovarian and testicular regulatory mechanisms coexist. This fish species has recently been used to describe the biological effects of contaminated marine sediments in light of its importance as a commercial food and its use as sentinel fish for environmental studies and monitoring [5]. The gilthead seabream is common in the Mediterranean Sea and, due to its euryhaline and eurythermal habits, the species is found in both marine and brackish water environments such as coastal lagoons and estuarine areas, particularly during the initial stages of its life cycle [22]. Moreover, the production of gilthead seabream in marine farms in the Mediterranean Sea is an industry with a promising future, whose current economic value is also significant, particularly in Spain, where turnover reached 88.8 million Euros in 2009 [23]. Levels of some xenobiotics are much higher in the Mediterranean Sea than in other seas and oceans [24], since, among other reasons, it has limited exchange of water with the Atlantic Ocean, and it is surrounded by some of the most heavily populated and industrialized countries in the world [25]. The reproductive cycle of gilthead seabream males is divided into four stages: spermatogenesis (SG), spawning, post-spawning and resting [20]. During the spermatogenesis stage all the different germ cell types develop from a testicular area, mainly formed by spermatogonia stem cells and cysts of primary spermatogonia. In fish, spermatogenesis occurs in a cystic structure in which all germ cells develop synchronously surrounded by a cohort of Sertoli cells, which nurse one germ cell type at a time [20,26]. During the spermatogenesis process, the germ cells reduce their chromosome content by meiosis and differentiate into spermatozoa. Once the spermatozoa are formed, they are released from the germinal epithelium into the lumen of the tubules together with the seminal fluid produced by Sertoli cells, where they remain until spawning. Afterwards, in the efferent duct system, spermatozoa are capacitated to fertilize the eggs. Once they make contact with seawater, the change in the osmolarity of the medium induces the motility of the spermatozoa and their final maturation [27].

In the gilthead seabream, and during the reproductive cycle, the levels of E_2_, T and 11KT [28], as well as the gene expression and production of several cytokines [19] vary. Interestingly, in male gilthead seabream specimens, E_2_ serum levels increase after the spawning stage when a massive infiltration of acidophilic granulocytes (AGs, the professional phagocytes in the gilthead seabream) into the gonad takes place [29,30,31], although this cell type does not express any of the three known nuclear ER, namely ERa, ERb1 and ERb2 [32]. Moreover, AG infiltration also occurs in the testis of specimens during the morphogenesis process [33]. These data, together with the expression pattern of cytokines and metalloproteinase (MMPs) by this cell type, suggested that AGs are essential for testicular tissue formation, remodeling and cell renewal [29]. We have recently reported that EE_2_ dietary intake disrupts spermatogenesis and promotes leukocyte infiltration in the gonad by up-regulating the expression of several genes involved in regulating leukocyte trafficking in the testis of SG stage fish [34]. Moreover, bath-exposure to EE_2_ might alter the capacity of gilthead seabream to appropriately respond to infection although this synthetic estrogen does not behave as an immunosuppressor [35]. Furthermore, it is known that the ability to respond to sex steroids or endocrine disruptors depends on the maturation stage of the fish [36].

In this framework, the present study tries to fill in some gaps in this knowledge by providing data about the effects of EE_2_ on the local immune regulation that takes place in the gonad in two different physiological stages of the reproductive cycle of a marine hermaphrodite fish, the gilthead seabream. Moreover, we analyze the gene expression profile of some molecules involved in the reproductive processes with the idea of using them as biomarkers of endocrine disruption. With this aim in mind, gilthead seabream specimens, in pre-SG (the stage just before to the spermatogenesis stage of the first reproductive cycle) and SG stages, were fed for 28 days with a pellet diet containing 5 μg EE_2_/g food, in order to determine whether EE_2_ promotes an estrogenic response by measuring the sperm quantity and quality, the serum levels of the main sex steroids and the gene expression of *vitellogenin* (*vtg*).

## 2. Results

### 2.1. EE_2_ Reduces the Stripped Volume of Seminal Fluid and Sperm Motility in Specimens in the Spermatogenesis Stage

In the SG stage, the dietary intake of EE_2_ decreased the stripped volume of seminal fluid and sperm motility but did not modify the stripped sperm concentration (Table 1). No data are presented for pre-SG specimens as no stripped sperm was obtained.

**Table 1:** Effects of the dietary intake of 5 μg 17α-ethynylestradiol (EE_2_)/g food during 7 and 28 days on volume of seminal fluid (mL), sperm concentration (cells/mL) and motility index at different exposure times. Data represent means ± SEM of six independent fish per group. * Asterisks denote statistically significant differences between treatment and control groups according to a Student *t* test (*p* ≤ 0.05).

	Sperm volume (mL)		Sperm concentration (cell/mL)		Sperm motility index	
Treatment (EE_2_)	7 days	28 days	7 days	28 days	7 days	28 days
**0 μg/g food**	0.63 ± 0.19	1.80 ± 0.38	(3.28 ± 0.76) × 10^9^	(6.46 ± 1.22) × 10^9^	2.08 ± 0.48	2.43 ± 0.23
**5 μg/g food**	0.25 ± 0.09	1.30 ± 0.79 *	(1.58 ± 0.97) × 10^9^	(2.57 ± 1.45) × 10^9^	1.46 ± 0.70	0.95 ± 0.48 *

### 2.2. EE_2_ Modifies Serum Sex Steroid Levels and Modulates the Gene Expression Profile of Some Steroidogenic Enzymes

**Figure 1:**
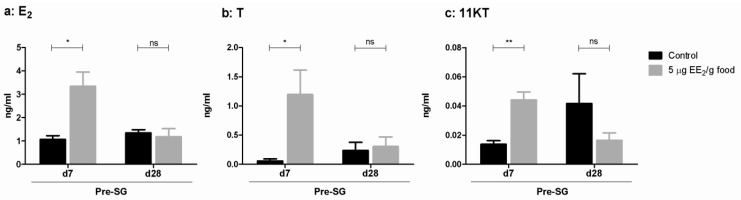
Modulation of serum sex steroid levels by dietary intake of EE_2_ exposure. 17β-estradiol (E_2_) (**a**), testosterone (T) (**b**) and 11-ketotestosterone (11KT) (**c**) serum levels were determined in gilthead seabream specimens in the pre-spermatogenesis (pre-SG) stage after the dietary intake of 0 (control) and 5 µg EE_2_/g food during 7 and 28 days. The serum sex steroid levels (ng/mL) from five to six fish/group were analyzed by ELISA. The asterisks denote statistically significant differences after Student *t* test between the untreated control group and the EE_2_ treated group at each time point. * *p <* 0.05 and ** *p <* 0.01. ns, not significant.

We have previously demonstrated in SG specimens of gilthead seabream that 5 μg EE_2_/g food promotes an increase in E_2_ and T serum levels after 7 days of treatment and a decrease in T and 11KT levels after 28 days [29]. Moreover, in pre-SG specimens, the dietary intake of 5 μg EE_2_/g food increased the E_2_ (Figure 1a), T (Figure 1b) and 11KT (Figure 1c) serum levels after 7 days of treatment, but did not significantly modify the same sex steroid levels at the end of the experiment. 

**Figure 2:**
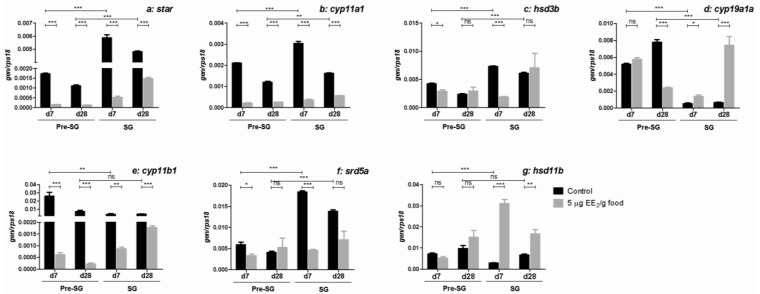
EE_2_ modulates the expression of genes coding for steroidogenic-relevant molecules in the gonad. Specimens at both pre-SG and SG stage were treated with 0 (control) and 5 µg EE_2_/g food during 7 and 28 days. Afterwards, the mRNA levels of *star* (**a**); *cyp11a1* (**b**); *hsd3b* (**c**); *cyp19a1a* (**d**); *cyp11b1* (**e**); *srd5a* (**f**) and *hsd11b* (**g**) were determined in the gonad by real-time reverse transcription polymerase chain reaction (RT-PCR). Total RNA was obtained after pooling the same amount of mRNA from five to six fish/group. Data represent means ± S.E.M. of triplicates of the same pooled sample. The asterisks denote statistically significant differences after Student *t* test between: (i) the untreated control group and the EE_2_ treated group at each time point and spermatogenic condition and (ii) the untreated control groups of the two spermatogenic conditions within the same sampling date. * *p <* 0.05, ** *p <* 0.01 and *** *p <* 0.001. ns, not significant.

We next investigated the gonadal gene expression of several steroidogenic enzymes (Figure 2). First, it was observed that the gene expression levels of steroidogenic acute regulatory protein (*star*) (Figure 2a), cholesterol side chain cleavage cytochrome P450 (*cyp11a1*) (Figure 2b), 3β-hydroxysteroid deshydrogenase (*hsd3b*) (Figure 2c) and 5α reductase (*srd5a*) (Figure 2f) were higher in SG than in pre-SG specimens. In contrast, the gene expression levels of aromatase (*cyp19a1a*) (Figure 2d), steroid 11-beta-hydroxylase (*cyp11b1*) (Figure 2e), and 11β-hydroxysteroid deshydrogenase (*hsd11b*) (Figure 2g) were lower in SG specimens. EE_2_ inhibited the expression of *star* (Figure 2a), *cyp11a1* (Figure 2b) and *cyp11b1* (Figure 2e) after 7 and 28 days of exposure and the expression of *hsd3b* (Figure 2c) and *srd5a* (Figure 2f) only after 7 days, in both pre-SG and SG specimens. However, a differential regulation in the expression of *cyp19a1a* was observed (Figure 2d): It was inhibited in pre-SG specimens after 28 days of dietary intake of EE_2_ and up-regulated in SG specimens after 7 and 28 days of dietary intake. Moreover, EE_2_ did not modify the expression levels of *hsd11b* (Figure 2g) in pre-SG specimens, but increased its expression in SG specimens at both sampling times. 

### 2.3. EE_2_ Increases the Expression Profile of the Hepatic *vtg* Gene

**Figure 3:**
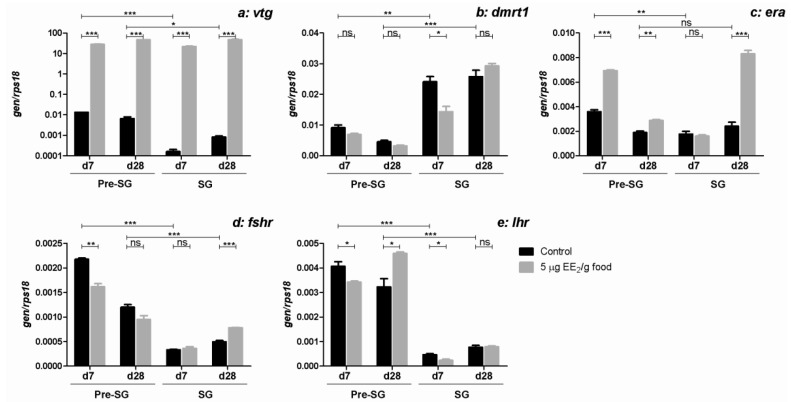
EE_2_ promotes an estrogenic response and modulates the expression of genes coding for hormone receptors. Specimens at both pre-SG and SG stages were treated with 0 and 5 µg EE_2_/g food during 7 and 28 days. Afterwards, the mRNA levels of *vtg* were determined in the liver (**a**) and the mRNA levels of *dmrt1* (**b**); *era* (**c**); *fshr* (**d**) and *lhr* (**e**) in the gonad by real-time RT-PCR. Total RNA was obtained after pooling the same amount of mRNA from five to six fish/group. Data represent means ± SEM of triplicates of the same pooled sample. The asterisks denote statistically significant differences after Student *t* test between: (i) the untreated control group and the EE_2_ treated group at each time point and spermatogenic condition and (ii) the untreated control groups of the two spermatogenic conditions within the same sampling date. * *p <* 0.05; ** *p <* 0.01 and *** *p <* 0.001. ns, not significant.

The expression of *vtg*, a gene induced by activation of nuclear ER [37], was slightly higher in pre-SG than in SG specimens. Moreover, the *vtg* expression levels were significantly up-regulated in the liver at both reproductive stages and EE_2_ exposure times assayed (Figure 3a).

### 2.4. EE_2_ Modulates the Expression of Testicular Specific Protein, Dmrt1, and Some Hormone Receptor Genes in the Gonad

As expected, expression of the gene that codes for the testicular specific protein, double sex-and mab3-related transcription factor 1 (*dmrt1*) was higher in the gonad of SG than in pre-SG specimens (Figure 3b). EE_2_ decreased the *dmrt1* expression levels in SG specimens after 7 days of exposure, but the effect disappeared after 28 days. No significant changes were observed in the *dmrt1* expression levels in pre-SG specimens after 7 or 28 days of exposure. 

Interestingly, the mRNA expression levels of estrogen receptor α (*era*) (Figure 3c), follicle stimulating hormone (FSH) receptor (*fshr*) (Figure 3d) and luteinizing hormone (LH) receptor (*lhr*) (Figure 3e) were higher in the gonad of pre-SG than SG specimens except for the *era* levels at day 28 of treatment. EE_2_ increased the *era* expression levels in the gonad of pre-SG specimens after 7 and 28 days or only after 28 days of exposure in SG (Figure 3c). In pre-SG specimens, EE_2_ decreased the *fshr* (Figure 3d)and *lhr* (Figure 3e) expression levels after 7 days of exposure, but increased *lhr* expression after 28 days (Figure 3e). Nevertheless, in SG specimens, *fshr* expression levels increased only after 28 days of EE_2_ dietary intake (Figure 3d), while the *lhr* expression levels were slightly lower after 7 days of EE_2_ exposure (Figure 3e). 

### 2.5. EE_2_ Modifies the Gene Expression of Molecules Relevant in the Immune Response in the Gonad

**Figure 4:**
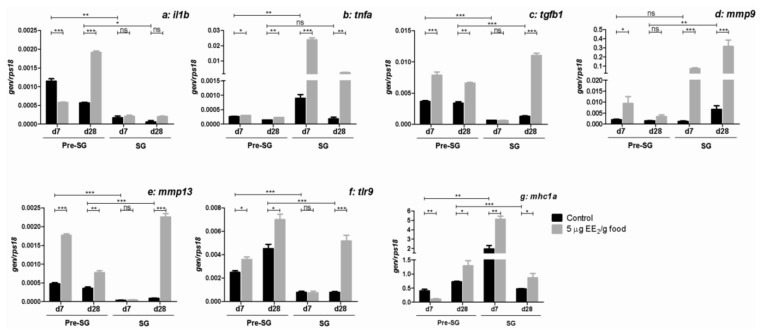
EE_2_ modulates the expression of genes coding for immune-relevant molecules in the gonad. Specimens at both pre-SG and SG stage were treated with 0 and 5 µg EE_2_/g food during 7 and 28 days. Afterwards, the mRNA levels of *il1b* (**a**); *tnfa* (**b**); *tgfb1* (**c**); *mmp9* (**d**); *mmp13* (**e**); *tlr9* (**f**); and *mhc1a* (**g**) were determined in the gonad by real-time RT-PCR. Total RNA was obtained after pooling the same amount of mRNA from five to six fish/group. Data represent means ± SEM of triplicates of the same pooled sample. The asterisks denote statistically significant differences after Student *t* test between: (i) the untreated control group and the EE_2_ treated group at each time point and spermatogenic condition and (ii) the untreated control groups of the two spermatogenic conditions within the same sampling date. * *p <* 0.05; ** *p <* 0.01 and *** *p <* 0.001. ns, not significant.

**Figure 5:**
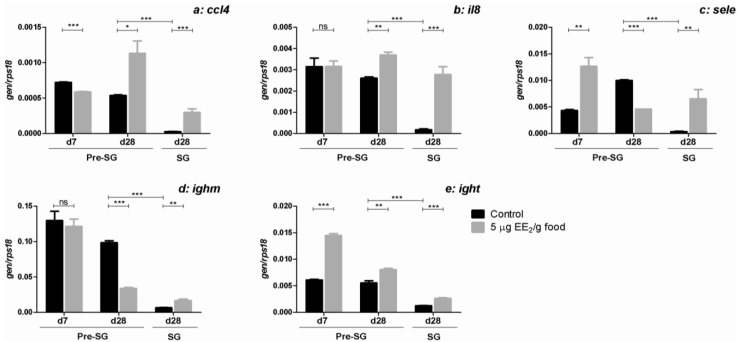
EE_2_ modulates the expression of genes involved in regulating leukocyte trafficking and lymphocytes B markers. Specimens at both pre-SG and SG stage were treated with 0 and 5 µg EE_2_/g food during 7 and 28 days and 28 days, respectively. Afterwards, the mRNA levels of *ccl4* (**a**); *il8* (**b**); *sele* (**c**); *ighm* (**d**) and *ight* (**e**) were determined in the gonad by real-time RT-PCR. Total RNA was obtained after pooling the same amount of mRNA from 5 to 6 fish/group. Data represent means ± SEM of triplicates of the same pooled sample. The asterisks denote statistically significant differences after Student *t* test between: (i) the untreated control group and the EE_2_ treated group at each time point and spermatogenic condition and (ii) the untreated control groups of the two spermatogenic conditions at day 28. * *p <* 0.05; ** *p <* 0.01 and *** *p <* 0.001. ns, not significant.

To explore the local immune regulation that occurred in the gonad, we analyzed the expression of genes coding for several pro- and anti-inflammatory cytokines, MMPs, molecules related with pathogen recognition, antigen presentation, leukocyte recruitment, and B lymphocytes markers (Figure 4 and Figure 5). Interestingly, almost all of the immune-related genes analyzed showed higher expression levels in pre-SG specimens than in SG, except those for tumor necrosis factor α (*tnfa*) (Figure 4b), matrix metalloproteinase (*mmp*) 9 (Figure 4d) and major histocompatibility complex I α protein (*mhc1a*) (Figure 4g), which were more highly expressed in SG specimens on at least one of the times analyzed.

EE_2_ was seen to differently modulate the expression levels of these immune-related genes in pre-SG and SG specimens. Thus, EE_2_ inhibited the expression level of interleukin 1β (*il1b*) after 7 days and increased it after 28 days in pre-SG specimens, while no significant differences were observed in SG specimens (Figure 4a). Moreover, the expression levels of *tnfa* (Figure 4b) and *mmp9* (Figure 4d) increased after EE_2_ exposure in both pre-SG and SG specimens, the increase being more pronounced in SG specimens. In contrast, the expression levels of transforming growth factor β1 (*tgfb1*) (Figure 4c), *mmp13* (Figure 4e) and toll-like receptor (*tlr*) 9 (Figure 4f) were increased by EE_2_ in pre-SG specimens at both times analyzed, and only after 28 days of exposure in SG specimens. As regards to the gene related with antigen presentation that codes for MHC Iα protein, named *spau-UAA* following the accepted nomenclature [38], EE_2_ increased the *mhc1a* mRNA levels in both pre-SG and SG specimens at both times analyzed, except after 7 days of exposure in pre-SG specimens where a decrease was observed (Figure 4g).

We previously demonstrated that the dietary intake of EE_2_ promoted an up-regulation in the gonad of the genes coding for CC chemokine ligand (*ccl4*), CXC chemokine interleukin 8 (*il8*) and leukocyte adhesion molecule E-selectine (*sele*), and the B lymphocyte markers, heavy chain of immunoglobulin M (*ighm*) and heavy chain of immunoglobulin T (*ight)*, in SG specimens after 7, 14 and 21 days of exposure, which occurred simultaneously with an infiltration of AGs and lymphocytes [34]. Here, we explore the differential regulation by EE_2_ of the expression of *ccl4* (Figure 5a), *il8* (Figure 5b), *sele* (Figure 5c), *ighm* (Figure 5d) and *ight* (Figure 5e), in pre-SG and SG specimens, after 7 and 28 days of exposure. As mentioned above, the expression levels of all these genes were higher in pre-SG than in SG specimens (Figure 5 and [34]). Similarly to what was previously observed after 7 days of EE_2_ exposure in SG specimens, EE_2_ increased the expression levels of all these genes in the gonad of SG specimens after 28 days. Nevertheless, in pre-SG specimens, EE_2_ modulated the expression levels of these genes in a different way. Thus, although *ccl4*, *sele* and *ight* expression levels increased after certain times of EE_2_ exposure, the expression levels of *ccl4* decreased and the transcription of *il8* and *ighm* were unchanged after 7 days of EE_2_ exposure. Moreover, *sele* and *ighm* expression levels fell after 28 days of EE_2_ exposure.

## 3. Discussion

EE_2_ is an environmental estrogen considered as an endocrine-disrupting compound (EDC) with strong estrogenic effects and a widespread presence in the aquatic environments [8,10]. Fish represent the animal group most affected by EDC exposure since they are continuously and directly exposed to these contaminants. Most authors agree that EE_2_ promotes an immature stage of the male gonads by blocking their development or by inducing the ablation of post-meiotic germ cells when immature fish or spermatogenically active fish are treated, respectively [39,40]. Moreover, in gonochoristic fish species, a widely observed effect of estrogenic compounds is the modification of sperm quality, sex steroid levels and hepatic Vtg production [37,41]. However, little is known about these effects in hermaphroditic fish species.

In the gilthead seabream, a hermaphroditic protandrous seasonal breeder, the spermatogenesis process proceeds in the testicular area, where it is orchestrated by high androgen levels; however, an increase in endogenous E_2_ levels coincides with spawning. EE_2_ promotes an estrogenic response, as seen from the increase in *vtg* gene expression levels in pre-SG and SG specimens. Interestingly, E_2_ serum levels increased after 7 days of exposure in both pre-SG and SG [34] specimens. However, T and 11KT levels differed between pre-SG and SG specimens, they both increased in pre-SG specimens after 7 days of exposure and decreased in SG after 28 days, probably due to the fact that T and 11KT serum levels were already very high in SG specimens compared with pre-SG specimens [34]. Moreover, exogenous E_2_ treatment of spermatogenically active males accelerated the final events of spermatogenesis and inhibited the proliferation of spermatogonia in early stages, promoting a post-spawning stage [42] which coincided with the decrease in sperm quality (volume of seminal fluid and the motility index) observed. No development of the ovary has been observed in gilthead seabream after 1 month of E_2_ [42] or EE_2_ [34] treatments, although primary oocytes appeared in the protandrous male black porgy after 5 months of E_2_ treatment [43].

Looking at the transcript regulation of the most relevant steroidogenic molecules involved in their production, we found that EE_2_ down-regulated the transcripts of *star*, *cyp11a1*, (two molecules that are synthesized rapidly in response to acute tropic hormone stimulation [44]), and *hsd3b*, *cyp11b1* and *srd5a* (steroidogenic molecules involved in androgen production) in the gonad of both pre-SG and SG specimens at all time points analyzed, while the *hsd11b* (steroidogenic enzyme involved in androgen production) and *cyp19a1a* (steroidogenic enzyme involved in estrogen production) expression levels were up-regulated in the gonad of SG males. Interestingly, the *cyp19a1a* was down-regulated in the gonad of pre-SG males. In the gilthead seabream, *cyp19a1a* expression gradually increased during the spermatogenesis and spawning stages, reaching a maximum at post-spawning. The serum levels of E_2_ increased progressively with each reproductive cycle [28]; moreover, during the second reproductive cycle the expression of *cyp19a1a* reached a higher level than during the first reproductive cycle [19]. These data, together with our present data, suggest that there is a reciprocal action between the estrogen serum levels and the expression of *cyp19a1a* gene in the SG specimens, while this mechanism is not effective in pre-SG specimens. This hypothesis would explain why, in the gilthead seabream, E_2_ seems to be essential for the renewal of the testis during the two first reproductive cycles [20,42], although high levels of this hormone are also needed in the sex change process that occurs at the beginning of the third reproductive cycle [45].

To assess whether any testicular reproductive parameters could be used as markers of endocrine disruption in gilthead seabream, the gene expression levels of the testicular specific protein, *dmrt1* and of some hormone receptor genes were analyzed in both stages of the reproductive cycle. In mammals, the depletion of Dmrt1 gene expression led to the loss of mitotic germ cells, which had precociously entered meiosis [46]. In gilthead seabream males, *dmrt1* gene expression was down-regulated at the end of the second reproductive cycle and the beginning of sex change [21]. Moreover, upon short-term estrogenic treatment, the testicular area of the gonad was depleted of pre-meiotic germ cells and showed an increase in spermatozoa [34]. Our data related to *dmrt1* expression levels in pre-SG and SG specimens explain these observations, as the *dmrt1* expression levels were not affected by the dietary intake of EE_2_ in pre-SG specimens, while in SG specimens, EE_2_ was seen to lower *dmrt1* expression levels after 7 days, although this effect had disappeared by the end of the treatment. 

Male germ cell development is regulated by the brain pituitary axis, which has evolved in vertebrates as a hormonal master control system over spermatogenesis and reproduction in general. Within this system, the pituitary gonadotropins, LH and FSH, play pivotal roles by regulating testis functions via their respective cognate receptors, LH receptor (LHR) and FSH receptor (FSHR). However, gonadal steroids and other agents that bind or prevent binding to steroid hormone receptors also regulate testicular functions [47]. In fact, in mammals, estrogens regulate testicular steroidogenesis acting though ERα [47]. Our data show that in the gilthead seabream, EE_2_ treatment increased the expression of *era* gene in the gonad of pre-SG and SG fish, although at different times. We have previously recorded increases in *era* gene expression upon E_2_ treatment in endothelial cells and macrophages *in vitro* [32,48] and upon EE_2_-bath exposure in head-kidney leukocytes *in vivo* [35]. Although *era* gene expression was seen to have increased in all the analyses carried out and could well be used as biomarker of endocrine disruption [49], the magnitude of the response differed from that observed for *vtg* gene expression and the time at which the effect appear became evident varied between stages and between tissues. Furthermore, these differences suggest changes in the sensitivity to estrogens during sexual maturation and point to the need for further studies to clearly determine life stages that are susceptible to estrogenic pollutants in fish. Moreover, EE_2_ dietary intake during 7 days down-regulated the *fshr* and *lhr* expression levels, while EE_2_ dietary intake during 28 days up-regulated the *fshr* expression level in SG specimens and the *lhr* expression levels in pre-SG specimens. Interestingly, all these data agree with the disruption of spermatogenesis and the recrudescence of the testicular area of the gonad and the non-induction of the sex change previously observed in SG gilthead seabream gonad upon EE_2_ dietary intake [34].

A relevant role for immune molecules in the regulation of spermatogenesis and/or steroidogenesis has been described in various vertebrates including fish [19,50]. In the gilthead seabream, the testis undergoes abrupt morphological changes especially after spawning, including a massive infiltration of AGs [20,21,29,51]. AGs are produced in the head-kidney and when they infiltrate the testis, they show heavily impaired functions [52]. Interestingly, the expression of genes coding for pro-inflammatory and anti-inflammatory mediators, innate immune receptors, lymphocyte receptors, anti-bacterial and anti-viral proteins and molecules related to leukocyte infiltration show a testicular pattern that depends on the reproductive stage of the gilthead seabream specimens [19] and which guarantees and modulates reproductive functions. In addition, endogenous increases of E_2_ in serum are correlated with AG infiltration into the gonad after spawning [29], while the dietary intake of EE_2_ by SG specimens of gilthead seabream induces the recruitment of AGs and B lymphocytes and up-regulates the expression of genes coding for molecules involved in leukocyte trafficking [34]. Moreover, specimens bath-exposed to EE_2_ show alterations in their capacity to appropriately respond to infection [35]. Although, EE_2_ modulates the expression pattern of immune molecules in gilthead seabream macrophages, which are known to be a key cell type in the immune-modulatory role played by E_2_ in the gilthead seabream gonad [32,35], little is known about the effects of EE_2_ and other environmental estrogens on the gene expression of immune-relevant molecules in the gonad of fish in general.

In the gilthead seabream, EE_2_ promotes an increase in the gonadal transcripts of the pro-inflammatory cytokines, *il1b* and *tnfa*, and the anti-inflammatory cytokine, *tgfb1*, although the response differs between pre-SG and SG specimens. These increases could be correlated with the decrease in androgen production and suggest, as occurs in mammals [53], that these cytokines are involved in testicular steroidogenesis; however, further studies are needed to confirm this observation. A similar conclusion was reached for the goldfish testis, in which a heterologous recombinant IL1β, murine IL1β, inhibited basal and human chorionic gonadotrophin-stimulated T production [54]. *Mmp9* and *mmp13* gene expression in the testis of gilthead seabream suggests a pivotal role for them in the regulation of the testicular physiology and, in particular, in the organization of the cysts during spermatogenesis and post-spawning, as well as in AG infiltration [55]. EE_2_ dietary intake promotes the transcription of *mmp9* and *mmp13* genes in the gonad of both pre-SG and SG specimens, which concords with the induction of the post-spawning stage and AG infiltration upon estrogen (E_2_ or EE_2_) exposure [34,42,55]. TLRs play important roles in the innate immunity of the male mammalian reproductive tract [56]. Although several *tlr* gene sequences have been reported in gilthead seabream, *tlr9* is the only one which is expressed in the gonad [19]. Our data show that the dietary intake of EE_2_ increases the expression of *tlr9* and *mhc1a* genes in the gonad of both pre-SG and SG fish, suggesting that this estrogenic pollutant stimulates the ability of the gonad to recognize and respond to pathogens. This is important when we consider that there are some pathogens that use the gonads to be transmitted to the next generations or to other animals [57]. However, further studies are needed to clearly determine the ability of the gonad to respond to gonad invasive pathogens under estrogenic pollutant conditions.

Finally and concerning the expression of genes that code for molecules involved in leukocyte recruitment (AGs and lymphocytes) into the gonad [29,58], and for B lymphocyte markers (IgM and IgT), higher expression levels were observed in the control group of pre-SG than in the control group of SG specimens [34]. Moreover, the EE_2_ dietary intake promotes bigger changes in most of those genes in pre-SG than in SG specimens. Probably, these differences in the level of gene expression observed between pre-SG and SG specimens resulted in differences in the leukocyte influx into the gonad in response to EE_2_.

## 4. Experimental Section

### 4.1. Animals and Experimental Design

Healthy specimens of gilthead seabream (Actinopterygii, Perciformes, Sparidae) were bred and kept at the Centro Oceanográfico de Murcia (Instituto Español de Oceanografía, Mazarrón, Murcia, Spain). 

The experiment was performed using 30 pre-SG specimens (June, with a body weight of 110 ± 20 g, 14-months old) and 30 SG specimens (November, with a body weight of 405 ± 25 g, 19 months old) of gilthead seabream males. The fish were kept in 2 m^3^ tanks with a flow-through circuit, suitable aeration and filtration system and natural photoperiod. The water temperature ranged from 14.6 to 17.8 °C. The environmental parameters, mortality and food intake were recorded daily. The EE_2_ was incorporated in the commercial food (44% protein, 22% lipids, Skretting, Spain) at doses of 0 (control) and 5 μg/g food, using the ethanol evaporation method (0.3 L ethanol/kg of food) as described elsewhere [59]. The specimens were fed *ad* *libitum* three times a day for 28 days and fasted for 24 h before sampling. Sampling was carried out after 7 and 28 days of EE_2_ exposure (*n =* 6 fish/group). For this, specimens were anesthetized with 40 µL/L of clove oil and the urogenital pore was dried before collecting sperm as described below. The specimens were then decapitated, weighed, and the livers and gonads were removed and processed for gene analysis, as described below. Serum samples from trunk blood were obtained by centrifugation and immediately frozen and stored at −80 °C until use. The experiments complied with the Guidelines of the European Union Council (86/609/EU), the Bioethical Committee of the University of Murcia (Spain) and the Instituto Español de Oceanografía (Spain) for the use of laboratory animals.

### 4.2. Measurement of the Volume of Seminal Fluid and Sperm Concentration and Motility

Stripped sperm was obtained by gentle abdominal massage, collecting and measuring the sperm in the genital pore with a syringe as the semen flowed out (urine-contaminated samples were discarded). The total semen from six fish of each group was used immediately to determine cell concentration and motility. To determine the sperm concentration, semen was diluted in 1% formol (Panreac) and 5% NaHCO_3_ (Sigma) in water at a ratio of 1:400 and the spermatozoa were counted using a Newbauer chamber. Motility was analyzed activating 1 μL of sperm (diluted on Ringer 200 mOsm solution at the optimal dilution of 1:5) with 20 μL of seawater [60]. The duration of sperm motility was determined by measuring the time elapsing between the initiation of sperm motility and the cessation of cell displacement using a light microscope at 400× magnification. The motility index was expressed on a relative scale of 0 to 5 [61].

### 4.3. Analytical Techniques

Serum levels of E_2_, T, and 11KT were quantified by ELISA following the method previously described [62]. Steroids were extracted from 20 μL of serum in 0.6 mL of methanol (Panreac). The methanol was then evaporated at 37 °C and the steroids were resuspended in 400 µL of reaction buffer [0.1 M phosphate buffer with 1 mM EDTA (Sigma), 0.4 M NaCl (Sigma), 1.5 mM NaN3 (Sigma) and 0.1% albumin from bovine serum (Sigma)]. Then, 50 µL were used in each well so that 2.5 µL of serum were used in each well for all the assays. E_2_ and T standards were purchased from Sigma-Aldrich. The 11KT standard, mouse anti-rabbit IgG monoclonal antibody (mAb), and specific anti-steroid antibodies and enzymatic tracers (steroid acetylcholinesterase conjugates) were obtained from Cayman Chemical. Microtiter plates (MaxiSorp) were purchased from Nunc. A standard curve from 6.13 × 10^−4^ to 2.5 ng/mL (0.03–125 pg/well) was established in all the assays. Standards and extracted serum samples were run in duplicate. The lower limit of detection for all the assays was 12.21 pg/mL. The intra-assay coefficients of variation (calculated from duplicate samples) were 3.98% ± 0.57% for E_2_, 8.26% ± 1.33% for T, and 8.80% ± 1.68% for 11KT for the pre-SG specimens. Details on cross-reactivity for specific antibodies were provided by the supplier (0.01% of anti-11KT reacts with T; 2.2% of anti-T reacts with 11KT; and 0.1% of anti-E_2_ reacts with T).

### 4.4. Analysis of Gene Expression

Total RNA was extracted from liver and gonad fragments with TRIzol Reagent (Invitrogen, Barcelona, Spain) following the manufacturer’s instructions, and quantified with a spectrophotometer (NanoDrop, ND-1000). The RNA of six fish per group was pooled using the same amount of RNA from each specimen. The RNA was then treated with DNase I (amplification grade, 1 unit/μg RNA, Invitrogen, Barcelona, Spain) to remove genomic DNA traces that might interfere with the PCR reactions and the SuperScript III RNase H− Reverse Transcriptase (Invitrogen, Barcelona, Spain) was used to synthesize first strand cDNA with oligo-dT_18_ primer from 1 μg of total RNA, at 50 °C for 50 min. Real-time PCR performed with an ABI PRISM 7500 (Applied Biosystems, Madrid, Spain) using SYBR Green PCR Core Reagents (Applied Biosystems, Madrid, Spain) was then used to analyze the expression of the genes coding for (i) steroidogenesis-related molecules: steroidogenic acute regulatory protein (*star*), cholesterol side chain cleavage cytochrome P450 (*cyp11a1*), 3β-hydroxysteroid deshydrogenase (*hsd3b*), aromatase (*cyp19a1a*), steroid 11-beta-hydroxylase (*cyp11b1*), 5α reductase (*srd5a*), and 11β-hydroxysteroid deshydrogenase (*hsd11b*); (ii) the hepatic vitellogenin (*vtg*); (iii) the testicular specific protein, double sex-and mab3-related transcription factor 1 (*dmrt1*); (iv) hormone receptors: estrogen receptor α (*era*), follicle stimulating hormone (FSH) receptor (*fshr*) and luteinizing hormone (LH) receptor (*lhr*) and; (v) immune-relevant molecules: interleukin 1β (*il1b*), tumor necrosis factor α (*tnfa*), transforming growth factor β1 (*tgfb1*), matrix metalloproteinase (*mmp*) 9 and 13 (*mmp13*), toll-like receptor 9 (*tlr9*), major histocompatibility complex I α protein (*mhc1a*), CC chemokine ligand (*ccl4*), CXC chemokine interleukin 8 (*il8*), leukocyte adhesion molecule E-selectine (*sele*) and heavy chain of immunoglobulin M (*ighm*) and T (*ight*). For each mRNA, gene expression was normalized to the ribosomal protein S18 gene (*rsp18*) content in each sample using the comparative Ct method (2^−∆∆Ct^) (where Ct is a cycle threshold). The gilthead seabream specific primers used are shown in Table 2. In all cases, each PCR was performed in triplicate.

**Table 2:** Gene accession numbers and primer sequences used for gene expression analysis [63].

Gene	Accession Number	Name	Nucleotide sequence (5′→3′)
*star*	AM905934	F1 R1	ACATCGGGAAGGTGTTCAAG TCTCTGCAGACACCTCATGG
*cyp11a1*	FM159974.1	F R	CGCTGCTGTGGACATTGTAT CATCATGTCTCCCTGGCTTT
*hsd3b*	HS985587	F R	GGAGGACAAACTGGTGGAGG ACATTCTCCGTTCCGGTGAC
*cyp19a1a*	AF399824	F2 R2	CAATGGAGAGGAAACCCTCA ATGCAGCTGAGTCCCTGTCT
*cyp11b1*	FP332145	F R	GCTATCTTTGGACCCCATCA CTTGACTGTGCCTTTCAGCA
*srd5a*	AM958800	F R	TGCACTTTCGTGACTCTGCT TTTCGCACAAGACGTCCAGA
*hsd11b*	AM973598	F R	AGACATGGGCAACGAGTCAG TCCACATCTCCCTCCCACAT
*vtg*	AF210428	F1 R1	CTGCTGAAGAGGGACCAGAC TTGCCTGCAGGATGATGATA
*dmrt1*	AM493678	F R	GATGGACAATCCCTGACACC GGGTAGCGTGAAGGTTGGTA
*era*	AF136979	F R	GCTTGCCGTCTTAGGAAGTG TGCTGCTGATGTGTTTCCTC
*fshr*	AY587262	F2 R2	TCCCACTACGGATCCTCATC AACGGGAACAGTCAGTTTG
*lhr*	AY587261	F2 R2	ATACACGACCACGCATTCAA CGCCGGTAACTTCTTGAGAG
*il1b*	AJ277166	F2 R3	GGGCTGAACAACAGCACTCTC TTAACACTCTCCACCCTCCA
*tnfa*	AJ413189	FE1 RE3	TCGTTCAGAGTCTCCTGCAG CATGGACTCTGAGTAGCGCGA
*tgfb1*	AF424703	F R	AGAGACGGGCAGTAAAGAA GCCTGAGGAGACTCTGTTGG
*mmp9*	AM905938	F1 R1	GGGGTACCCTCTGTCGATTT CCTCCCCAGCAATATTCAGA
*mmp13*	AM905935	F R	CGGTGATTCCTACCCATTTG TGAGCGGAAAGTGAAGGTCT
*tlr9*	AY751798	F2 R2	GGAGGAGAGGGACTGGAT GATCACACCGTCACTGTCTC
*mhc1a*	AY292461	F R	CCAGAGCTTCCCTCAGTGTC CATCTGGAAGGTTCCATCGT
*ccl4*	AM765840	F1 R1	GCTGTGTTTGTGCTGATGCT GCTGGCTGGTCTTTTGGTAG
*il8*	AM765841	F2 R2	GCCACTCTGAAGAGGACAGG TTTGGTTGTCTTTGGTCGAA
*sele*	AM749963	F1 R1	GACAGTGAGCAGGCGTACAA ATCGCTTCATGATCCACACA
*ighm*	AM493677	F1 R1	CAGCCTCGAGAAGTGGAAAC GAGGTTGACCAGGTTGGTGT
*ight*	FM145138	F1 R1	TGGCAAATTGATGGACAAAA CCATCTCCCTTGTGGACAGT
*rps18*	AM490061	F R	AGGGTGTTGGCAGACGTTAC CTTCTGCCTGTTGAGGAACC

### 4.5. Calculation and Statistics

All data related to the stripped volume of seminal fluid, sperm concentration and motility index, sex steroid serum levels and gene expressions were analyzed by a Student *t-*test to determine differences between untreated control and the treated group for each time point. The critical value for statistical significance was taken as *p* ≤ 0.05. The asterisks mean: * *p <* 0.05; ** *p <* 0.01 and *** *p <* 0.001. All statistical analyses were carried out using the GraphPad Prism 5 program.

## 5. Conclusions

Our data demonstrate that the dietary intake of EE_2_ promotes an estrogenic response and modifies the expression pattern of steroidogenic molecules, cytokines and other immune-related molecules involved in different processes of testicular physiology, ranging from steroidogenesis to pathogen recognition. Interestingly, a developmental adjustment of the sensitivity to EE_2_ in the gilthead seabream gonad was observed, pointing to the need for further studies to clearly determine the life stages most susceptible to estrogenic pollutants in fish.

## Declaration

Genetic nomenclature used in this manuscript follows the guidelines of the Zebrafish Nomenclature Committee (ZNC) for fish genes and proteins and the HUGO Gene Nomenclature committee for mammalian genes and proteins.
